# In Situ Fabrication of Gradient Porous Layers on Sintered Metallic Substrates via Surface Friction Treatment

**DOI:** 10.3390/ma18184220

**Published:** 2025-09-09

**Authors:** Kun Yang, Shuai Zhang, Chenyang Xu, Shaoyang Zhao, Lei Shen

**Affiliations:** State Key Laboratory of Porous Metal Materials, Northwest Institute for Nonferrous Metal Research, Xi’an 710016, China

**Keywords:** functionally graded metallic materials, gradient layer, surface friction treatment, pore size distribution

## Abstract

This work pioneers the novel application of surface friction treatment (SFT) to sintered porous metals to fabricate gradient-structured functional surfaces. The results demonstrate that SFT significantly modifies surface pore architecture, with scanning velocity and normal load critically controlling gradient layer formation. Excessive parameters induce periodic surface cracking due to mechanical overloading. Post-treatment, the porous metal exhibits a 37.5% reduction in maximum pore size (24 μm to 15 μm) and substantially improved surface finish, with arithmetic mean roughness (Ra) decreasing from 23.8 μm to 4.3 μm (82% reduction). These surface enhancements significantly improve filtration functionality while providing a cost-effective alternative to conventional gradient layer fabrication methods.

## 1. Introduction

Functionally graded materials (FGMs) are heterogeneous materials characterized by gradients in composition and/or structure across multiple material components [1]. Recently, FGMs have garnered significant attention due to their superior strength–ductility synergy, high fracture toughness, and enhanced resistance to fatigue, wear, and corrosion, positioning them as promising materials at the forefront of research [2,3,4,5,6,7,8,9]. Consequently, substantial research efforts have focused on developing gradient structures within fully dense materials, exemplified by techniques such as Surface Mechanical Attrition Treatment [10,11,12], Surface Mechanical Grinding Treatment [13,14,15], and surface friction treatment [16,17,18].

Similar to their dense counterparts, gradient porous materials represent another class of functionally graded materials [19,20,21], with natural examples including bone and plant roots [22,23]. The gradient pore architecture enhances the performance of engineered porous materials for specific applications. For instance, in the effective purification and separation of industrial dust-laden gases, gradient powder-sintered porous tubes can effectively balance permeation flux and filtration accuracy [24].

Gradient powder-sintered porous tubes (commonly termed metallic membranes) typically comprise a macroporous support substrate and a thin microporous separation layer. Conventional fabrication involves initially forming the support body through cold isostatic pressing and sintering coarse metal powder. A microporous layer is subsequently applied by iterative coating, drying, and sintering cycles using fine metal or metal oxide powders. This methodology necessitates at least two distinct sintering stages, rendering the process inherently costly and time-intensive. Consequently, these limitations impede the broader industrial implementation of metallic membranes for microfiltration applications. In addition, during gradient structure fabrication, fine powder infiltration into the support typically forms a particle-permeated interlayer, introducing undesirable hydraulic resistance to the metallic membrane. In summary, traditional preparation methods face challenges: particle infiltration compromises material permeability, and repeated sintering steps substantially increase manufacturing costs [25,26,27,28].

Therefore, this study proposes a novel in situ process, inspired by fabrication methods used for dense functionally graded materials to create gradient porous layers on sintered porous metallic substrates via surface friction treatment. The deformation mechanism governing gradient porous structure formation during surface friction treatment was elucidated. Furthermore, the effects of process parameters on the surface morphology, pore structure, and surface roughness of gradient porous samples were systematically investigated.

## 2. Materials and Methods

### 2.1. Material and Surface Friction Treatment

**Figure 1:**
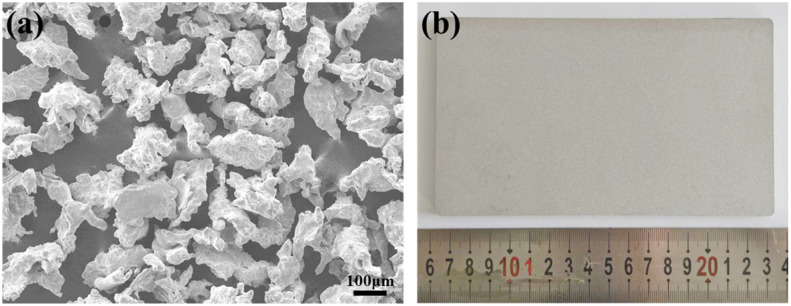
Morphology of 316L pre-alloyed powders (**a**) and sintered porous sample (**b**).

Water-atomized 316L stainless steel pre-alloyed powder (particle size: 80–150 μm, Figure 1a) was used to fabricate green porous flat plates (170 × 100 × 4 mm^3^) via cold isostatic pressing (180 MPa, 30 s hold time). Sintering was performed in a vacuum atmosphere (10^−2^ to 10^−3^ Pa) at 1250 °C for 2 h. The as-sintered sample is shown in Figure 1b.

**Figure 2:**
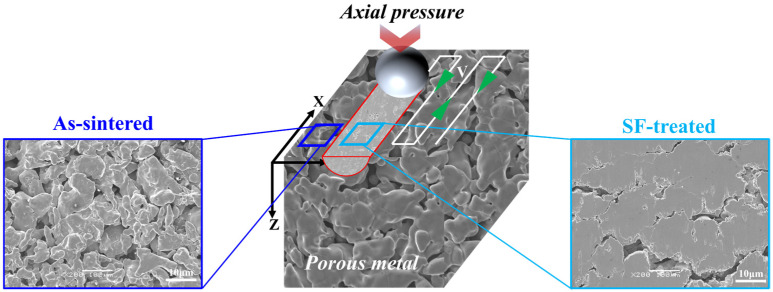
Schematic diagram of the graded porous metal preparation process using surface friction treatment. The green arrow in the figure indicates the path taken by the WC-Co ball.

**Figure 3:**
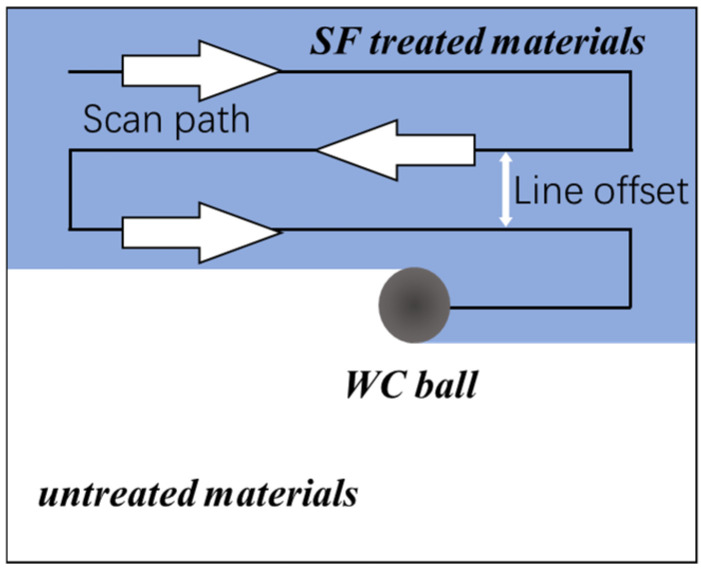
Schematic of the scanning strategy during surface friction treatment.

Surface friction treatment was conducted on a custom-built device using a ball-on-disc configuration, as illustrated in Figure 2. The sintered porous sample was mounted on the worktable and brought into contact with a spherical WC-Co ball (diameter: 10 mm). A controlled normal load (F) was applied by adjusting the vertical displacement of the ball. The ball then traversed the sample surface along the X-direction at a constant velocity (V). For single-line scans, the ball traversed the path once. For surface treatments, a serpentine raster scanning strategy was employed with a line offset of 100 μm, as shown in Figure 3. The scanning velocity (V) and normal load (F) were varied within the ranges of 30–250 mm/s and 30–250 N, respectively. The adopted parameter range derives from systematic experimental studies on porous titanium (Grade 2), 316L stainless steel, and Monel 400 systems.

### 2.2. Characterization

For the characterization of surface and cross-section morphology, smaller specimens were cut from the flat plate using wire electrical discharge machining, then cleaned with acetone, alcohol, and water; subsequently, these specimens were examined using scanning electron microscopy (SEM; Hitachi JSM-6460, Tokyo, Japan). For the determination of pore size distribution and gas flux, a circular sample with a 20 mm diameter was cut from the flat samples and cleaned and dried; then, these samples were analyzed using a pore size analyzer (GaoQ PSDA-30M, GaoQ Functional Materials Co., Ltd., Nanjing, China) according to ASTM F316. Nitrogen gas was used as the permeant for all gas flux and pore size measurements. The surface roughness Ra was characterized with a white light interferometer (KLA Tencor-Micro XAM, KLA, Milpitas, CA, USA). Triplicate tests were performed under each experimental condition to ensure data accuracy and reproducibility.

## 3. Results and Discussion

### 3.1. Morphology Evolution of Porous Samples

**Figure 4:**
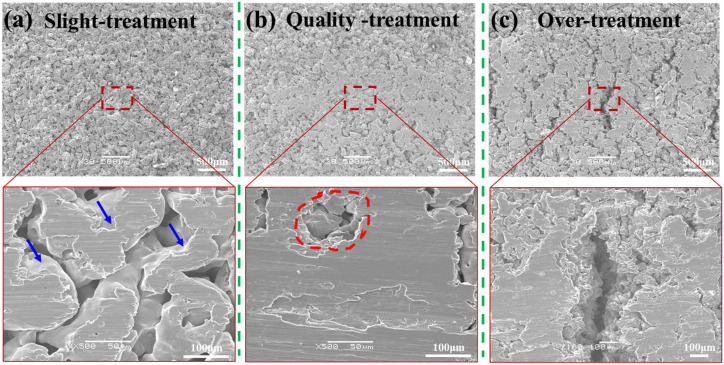
Representative surface morphology of the porous flat samples during surface friction treatment. (**a**) Slightly treated sample (V = 50 m/s, F = 50 N); (**b**) quality-treated sample (V = 100 m/s, F = 100 N); and (**c**) over-treated sample (V = 200 m/s, F = 200 N). The blue arrow indicates the original morphology of the powder particles. The region enclosed by the red dashed circle represents the pores formed in the material after processing.

Three representative surface morphologies and corresponding cross-section micrographs of porous samples that were subjected to single-line scanning treatment are shown in Figure 4. At a low scanning velocity, V, and low normal load, F, limited energy input preserved the original pore structure. As shown in Figure 4a, surface particles retained their morphology with only superficial wear (as indicated by the blue arrow in Figure 4a), while visible WC ball traces confirmed minimal disruption to subsurface pores.

When V and F reached optimal values (Figure 4b), sufficient energy input produced smooth surfaces with refined micropores, as indicated by the red circle. The original particle morphology was fully eliminated and replaced by a homogeneously deformed layer. Further increases in V and F (Figure 4c) caused excessive energy input, resulting in oversliding, severe deformation, and cracks propagating perpendicular to the scanning direction.

### 3.2. Processing Window and Deformation Mechanism

**Figure 5:**
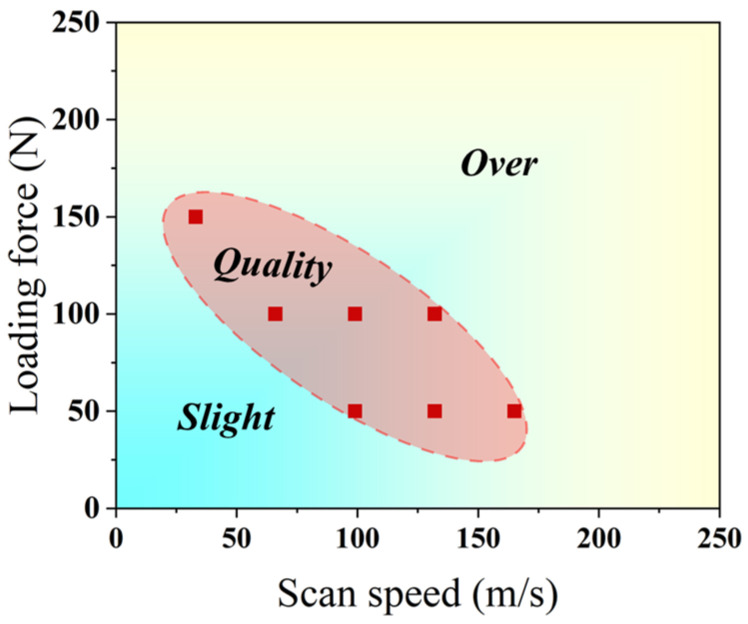
Processing window for gradient porous layer by surface friction treatment.

Based on the morphological analysis in Section 3.1, the processing window for surface friction treatment was established using the scanning velocity V and normal load F as controlling parameters (Figure 5). Three distinct regimes were identified: slight treatment, quality treatment, and over-treatment. The dashed region indicates suitable parameters for fabricating crack-free gradient porous layers. Cracking occurs when F exceeds ~160 N or V exceeds ~160 mm/s, as the excessive mechanical energy input surpasses the material’s fracture strength. 

**Figure 6:**
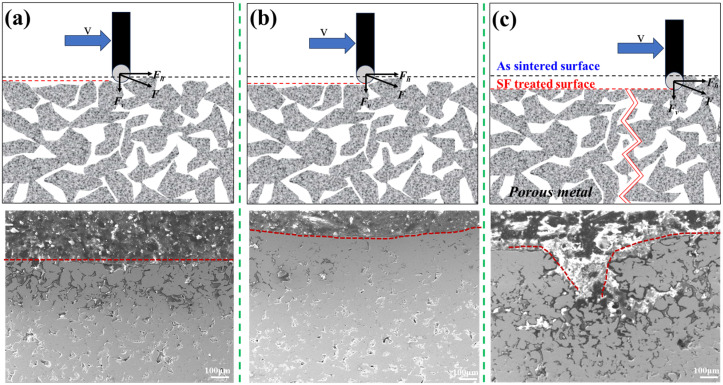
Schematic sketch of the deformation process during surface friction treatment and the representative cross-sectional SEM image of the samples. (**a**) Slightly treated sample; (**b**) quality-treated sample; and (**c**) over-treated sample. The red dashed line delineates the surface contour of the fabricated sample.

The gradient layer formation mechanism is illustrated in Figure 6. The applied normal force F resolves into the following:

1. A vertical component (F_v_) compressing the porous surface;
2. A horizontal component (F_h_) imparting shear deformation.

In slight treatment (Figure 6a), limited F_v_ and F_h_ cause minor surface compression without altering the pore structure through the depth. In quality treatment (Figure 6b), optimal F_v_ compresses the top ~200 μm layer while F_h_ polishes the surface, creating a gradient layer while preserving interconnected subsurface pores. When F_h_ exceeds the porous metal’s fracture strength (Figure 6c), cracks propagate within the gradient layer (Figure 6b), reaching dimensions of ~200 μm (width) × 400 μm (depth). 

Additionally, the cross-sectional analysis revealed compression of the porous sheet while maintaining interconnected internal pores, successfully generating a gradient porous layer atop the sintered powder substrate. These results indicate that the thickness of the gradient structure layer obtained through SBF treatment will fall within a reasonable range. Cracks will occur in an excessively thick gradient layer, which is beneficial for ensuring the material’s permeation flux [29,30].

### 3.3. Pore Architecture Evolution 

**Figure 7:**
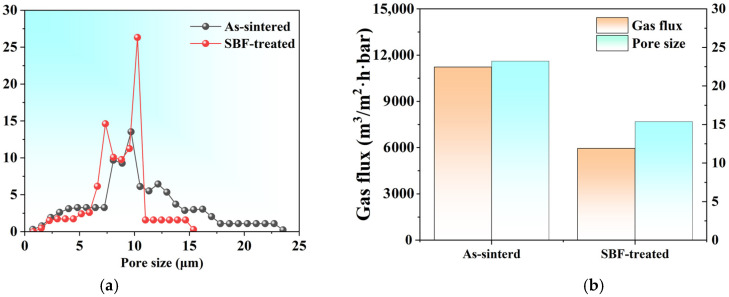
Pore size distributions (**a**), gas flux, and pore size (**b**) of porous flat samples before and after surface friction treatment.

Based on the above analysis, the quality treatment sample (F = 100 N, V = 100 m/s) was further characterized using pore size analysis. As shown in Figure 7a, the pore size distribution in the quality treatment sample exhibits a narrower distribution primarily ranging from 5 to 12 μm, which is different from the broad pore size distribution in the as-sintered porous sample (5–25 μm). At the same time, the maximum pore size of the sample decreased from 25 μm to 15 μm after surface friction treatment. The main reason for the narrowing of pore size distribution is the compression of surface pores and deformation of pore channels during the processing [31]. Although permeability decreases by ~40% (Figure 7b), the reduced pore size and narrower pore size distribution enable effective microfiltration of submicron particles from gas streams [32,33].

### 3.4. Surface Topography Modification

**Figure 8:**
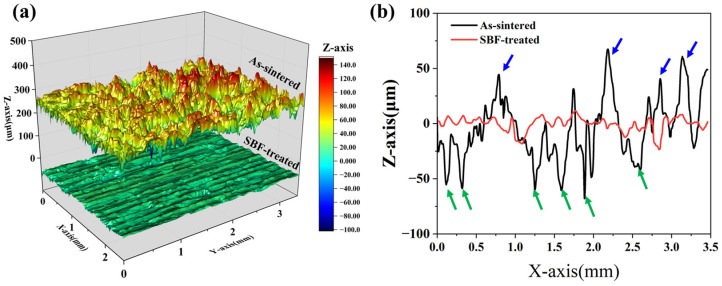
The 3D morphology (**a**) and 2D surface profiles (**b**) of as-sintered and SBF-treated porous samples. The blue and green arrows in the figure represent the peaks and valleys formed by the unevenness of the material surface, respectively.

As shown in Figure 8a, the surface morphologies of the porous material before treatment exhibit significant roughness and undulations, with distinct morphological features of the powder particles visible. The surface roughness of the as-sintered sample was 23.8 μm, which is consistent with the roughness of powder metallurgy porous materials reported in the previous literature [34,35]. After treatment, the surface of the flat porous samples became markedly smoother, and the surface roughness showed a sharp decline from 23.8 μm to ~4.3 μm. This can be associated with two facts. First, the original powder particle characteristics at the top surface of the sample were completely eliminated by the movement of the WC ball; thus, the number and intensity of peaks in the surface profile obviously decreased, as indicated by the blue arrows in Figure 8b. Second, the pore mouth, as shown in Figure 4b, is notably reduced in size, resulting in a significant decrease in valley values, as the green arrows indicate in Figure 8b.

Previous studies [34,36] demonstrate that rough surfaces of porous metallic filtration elements promote adhesion and filter cake formation of micrometer-sized particles. This necessitates frequent pulse-jet cleaning cycles and causes excessive operational pressure drops. In contrast, smooth-surfaced filtration elements—featuring gentler topographic variations—significantly reduce particle adhesion and deposition. Conventional fabrication of gradient porous metals requires sequential sintering of the support layer followed by deposition and re-sintering of the functional gradient layer [23,37,38]. This two-step approach incurs high manufacturing costs and prolonged processing times, hindering widespread adoption of metallic membranes for microfiltration. The surface friction treatment (SFT) developed in this work addresses these limitations by providing a single-step, cost-efficient route to fabricate gradient porous metallic materials optimized for micrometer-scale particle filtration.

## 4. Conclusions

Gradient porous layers were successfully fabricated in situ on sintered porous 316L substrates using surface friction treatment (SFT). Samples exhibiting slight treatment, quality treatment, and over-treatment characteristics were identified, and a processing window for SFT was established based on the scanning velocity and normal load. The morphology, pore structure, gas flux, and surface roughness of gradient samples were systematically analyzed and compared with those of as-sintered counterparts. The main conclusions are summarized as follows.

1. Crack-free gradient porous layers were achieved within optimal processing parameters (scanning velocity < 160 mm/s; normal load < 160 N). Exceeding these thresholds induced crack formation throughout the gradient layer.
2. Pore structure refinement occurred via surface compression during SFT, narrowing the pore size distribution and reducing maximum pore size from 25 μm to 15 μm. This decreased gas fluctuation by approximately 40%.
3. Surface roughness was significantly reduced from 23.8 μm (as-sintered) to ~4.3 μm (SFT-treated), resulting in markedly smoother surfaces.
4. The reduction in surface pore size and optimization of roughness resulted in better surface functionality and cost advantages for the gradient porous material obtained through SBF treatment.

## Data Availability

The original contributions presented in this study are included in the article. Further inquiries can be directed to the corresponding author.
